# Accuracy of telephone triage in patients suspected of transient ischaemic attack or stroke: a cross-sectional study

**DOI:** 10.1186/s12875-020-01334-3

**Published:** 2020-12-05

**Authors:** Daphne C. Erkelens, Frans H. Rutten, Loes T. Wouters, L. Servaas Dolmans, Esther de Groot, Roger A. Damoiseaux, Dorien L. Zwart

**Affiliations:** Department of General Practice, Julius Center for Health Sciences and Primary Care, University Medical Center Utrecht, Utrecht University, STR 6.131, P.O. Box 85500, 3508 GA Utrecht, The Netherlands

## Abstract

**Background:**

The Netherlands Triage Standard (NTS) is a widely used decision support tool for telephone triage at Dutch out-of-hours primary care services (OHS-PC), which, however, has never been validated against clinical outcomes. We aimed to determine the accuracy of the NTS urgency allocation for patients with neurological symptoms suggestive of a transient ischaemic attack (TIA) or stroke, with the clinical outcomes TIA, stroke, and other (neurologic) life-threatening events (LTEs) as the reference.

**Method:**

A cross-sectional study of telephone triage recordings of patients with neurological symptoms calling the OHS-PC between 2014 and 2016.The allocated NTS urgencies were derived from the electronic medical records of the OHS-PC. The clinical outcomes were retrieved from the electronic medical records of the patients’ own general practitioners. The accuracy of a high NTS urgency allocation (medical help within 3 h) was calculated in terms of sensitivity, specificity, positive and negative predictive values (PPV and NPV) with the clinical outcomes TIA/stroke/other LTEs as the reference.

**Results:**

Of 1269 patients, 635 (50.0%) received the diagnosis TIA/stroke (34.2% TIA/minor stroke, 15.8% major ischaemic or haemorrhagic stroke), and 4.8% other LTEs. For TIA/stroke/other LTEs, the sensitivity and specificity of the NTS urgency allocation were 0.72 (95%CI 0.68–0.75) and 0.48 (95%CI 0.43–0.52), and the PPV and NPV were 0.62 (95%CI 0.60–0.64) and 0.58 (95%CI 0.54–0.62).

**Conclusions:**

The NTS decision support tool used in Dutch OHS-PC performed poor to moderately regarding safety (sensitivity) and efficiency (specificity) in allocating adequate urgencies to patients with and without TIA/stroke/other LTEs.

**Trial registration:**

The Netherlands National Trial Register, identification number NTR7331 /Trial NL7134.

## Background

Prompt recognition of patients with a transient ischaemic attack (TIA) or stroke is crucial for timely initiation of therapeutic interventions to minimise the risk of (permanent) brain injury and recurrent stroke [1–6]. Previous studies showed that urgent diagnostic assessment of TIA and minor stroke patients followed by a timely start of stroke preventive treatment resulted in a tremendous decrease of the early stroke risk [1, 5, 7] with a reduction of recurrent stroke up to 80% within 3 months [1]. However, the detection of TIA, and to a lesser extent stroke, may be challenging because multiple other diseases like migraine with aura, seizures or syncope can mimic TIA or stroke [8–10]. Moreover, symptoms may be non-specific in TIA or stroke, notably vertebrobasilar insufficiency, and in the case of TIA, symptoms are often short lasting and already resolved by the time a patient seeks medical help [8, 11].

Patients with symptoms suggestive of TIA or stroke often contact the general practitioner (GP) first [12–15]. During evenings, nights and weekends such care is provided by the out-of-hours services in primary care (OHS-PC). At the OHS-PC, the initial contact is by telephone, and nurses perform triage while supervised by GPs [16]. The goal of telephone triage is to assess the severity of patients’ complaints and to link this to an adequate urgency allocation with corresponding response time to medical care. Telephone triage in the Netherlands is supported by a semi-automatic decision support tool called the ‘Netherlands Triage Standard’ (NTS). The NTS is a five-level triage tool, which was developed by an expert panel and derived from existing Dutch national telephone guidelines for primary care office hours, and the Manchester Triage System (MTS) [17, 18]. Based on the annual incidence of 0.006% of serious adverse events (SAEs) in the Dutch OHS-PC setting, the NTS is considered to be safe [19]. However, questions have been raised about the efficiency [16]. There was a clear increase in high urgency allocations since the implementation of the NTS in 2011 onwards, suggesting a low efficiency [20]. This was supported by the results of a national survey among GPs in 2016, showing that the vast majority believed telephone triage with the NTS resulted in unnecessary consultations and home visits [16, 21].

Most previous studies assessed the overall accuracy of triage decision support tools in emergency department (ED) settings, and only a few studies did this in the OHS-PC [22, 23]. Few studies focused on specific domains of patients (e.g. chest pain), some of which included clinical outcomes as the reference (e.g. acute coronary syndrome), yet, only in ED settings [24–31]. Comparable accuracy studies in primary care settings are limited; one study that assessed the overall accuracy of a telephone triage tool in primary care used a ‘surrogate’ reference created by the researchers themselves (e.g. hospital referrals or costs) [18, 22, 32–34]. The NTS urgency allocation, or the urgency allocation of other decision support tools for telephone triage in primary care settings, were never evaluated against the final clinical outcomes of patients as the reference.

We aimed to determine the accuracy of the NTS urgency allocation in patients calling the OHS-PC with symptoms suggestive of TIA or stroke, with presence or absence of the final clinical outcomes TIA, stroke and other (neurologic) life-threatening events (LTEs) as the reference.

## Methods

### Design and setting

We conducted a cross-sectional study in which we analysed real-life telephone triage recordings of nine OHS-PC locations in the vicinity of Utrecht, the Netherlands between 2014 and 2016. These OHS-PCs provide out-of-hours primary care for approximately 1,5 million people, handling 400,000 triage calls per year.

### Data collection

We evaluated patients with symptoms suggestive of TIA or stroke. The accuracy of NTS urgency allocation was assessed with the final clinical outcomes as the reference, that is, TIA, stroke and other (neurologic) life-threatening events (LTEs), e.g. intracranial haemorrhage. The triage recordings were selected in a two-step inclusion procedure, i.e. (i) selection based on the International Classification of Primary Care (ICPC) codes that are linked to the call and reflected our study domain (i.e. K89, K90, N17, N18, N19, N29, N89, N91), together with (ii) keywords in the OHS-PC electronic medical records suggesting TIA/stroke (e.g. neurological deficit, arm or leg weakness, face drooping, communication problem, visual problem, sensory disturbances and common synonyms) [35]. A detailed description of the ICPC codes, medical keywords, inclusion and exclusion criteria has been published elsewhere [36]. We selected a random sample of 2209 calls by using the Random Number Generator (RAND) function in Microsoft Excel. After a brief training and by means of a standardised case record form the triage calls were listened back and scored by 14 junior researchers. Two researchers from the study team (DCE and LTW) randomly checked one-third of all included calls. Patient and call characteristics, and assigned NTS urgencies were collected. From the patients’ own GPs we retrieved the final diagnosis, which was based on the discharge letter from the neurologist or the ED if the patient was referred for additional investigations. For patients who were not referred to the hospital we used follow-up data from the electronic medical records of GPs for up to 1 month to capture possible recurrence of TIA/stroke.

### NTS urgency allocation in day-to-day practice

Telephone triage with the NTS starts with a mandatory ‘ABCD’ check (i.e. airway, breathing, circulation, disability). In case of direct life-threatening situations, an ambulance will be sent immediately [37]. If there is no life-threatening situation, the triage nurse continues by choosing one out of the 56 main complaints within the NTS. Every main complaint consists of an algorithm composed of hierarchically ordered questions [18]. .One of these 56 main complaints is ‘neurological deficit’. After filling out the patient’s responses, the NTS will automatically generate an urgency level ranging from U0 to U5 which is linked to the response time within which a patient should receive medical help (see Table 1) [18, 38]. The NTS urgency may be scaled up or down by the triage nurse, often after first consulting the supervising GP [21]. The reason for overruling should be registered, but this is not a mandatory step to complete the NTS triage process.

**Table 1:** NTS levels of urgency

NTS Urgency level	Definition	Response time	Medical help
U0 – Resuscitation	Loss of vital functions	Immediately	Ambulance
U1 – Life threatening	Unstable vital functions	Within 15 min	Ambulance
U2 – Emergent	Vital functions in danger or organ damage	As soon as possible, within 1 h	Home visit by GP or appointment at OHS-PC
U3 – Urgent	Possible risk of damage, human reasons	A few hours (< 3 h)	Home visit by GP or appointment at OHS-PC
U4 – Non-urgent	Marginal risk of damage	24 h	Appointment at OHS-PC or telephone advice
U5 – Advice	No risk of damage	Advice, no time related	Telephone advice

*GP* General Practitioner, *NTS* Netherlands Triage Standard, *OHS-PC* Out-Of-Hours Services in Primary Care

### Difference between NTS urgency and final urgency

Besides the NTS urgency, which is automatically generated, we also evaluated the final urgency, which was defined as either the NTS urgency (if not changed) or the overruled NTS urgency.

In around 20% of all triage calls, the final urgency was unclear after re-listening the recordings in which it was evident that the triage nurse overruled the NTS urgency. This because the triage nurse did not notify the actual allocated urgency after overruling the NTS; e.g. the NTS urgency was U3, but in the audio recording the triage nurse tells the caller “I will sent an ambulance immediately” (U1)). Nevertheless, the urgency in the NTS system remained U3. A panel of three experienced GPs assessed calls in which the final urgency was unclear, blinded to the final diagnosis, and determined the final urgency (unanimously, or majority of votes after group discussion).

### Data analyses

The patients were dichotomised into a high (U1 and U2) and low (U3, U4 and U5) urgency group, and differences in characteristics between these groups were compared. We calculated the accuracy in terms of sensitivity, specificity, positive and negative predictive values of (i) the NTS urgency allocation and (ii) the final urgency allocation (including overruled NTS urgencies), with the clinical outcomes TIA/stroke/LTEs as the reference. For the accuracy calculations we considered for TIA/minor stroke case the urgencies U1, U2 and U3 as adequate, and for major stroke and other LTEs the urgencies U1 and U2. Finally, we compared the baseline characteristics of patients in whom we could retrieve the final diagnosis with those in whom we could not, to assess potential selection bias. Statistical analyses were performed using SPSS version 25.0 (IBM Corp., Armonk, NY, USA).

## Results

### Group characteristics

**Fig. 1:**
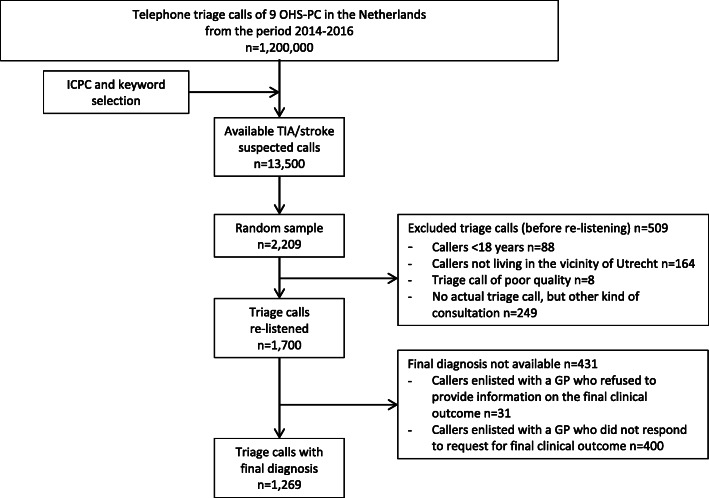
Flowchart study population

We included 1269 patients of whom a final diagnosis could be obtained (see Fig. 1). The median age was 72.0 (IQR 57.0–83.0) years, and 56.9% were female. The NTS allocation of high (U1 and U2) and low (U3, U4 and U5) urgencies was equally distributed between men and women (see Table 2).

**Table 2:** Characteristics of 1269 patients with symptoms suggestive of TIA or stroke calling the OHS-PC

	High NTS urgency ***n*** = 770 (60.7%)	Low NTS urgency ***n*** = 499 (39.3%)	***P***-value^**#**^
**Patient characteristics:**			
Median age in years (IQR)	73.5 (59.0–84.0)	69.0 (55.0–82.0)	< 0.001
Female sex	441 (57.3)	281 (56.3)	0.736
Family history of CVD (*n* = 36)	14 (82.4)	13 (68.4)	0.451^&^
**Medical history:**			
Cardiovascular disease (*n* = 882)	421 (79.1)	259 (74.0)	0.076
TIA (*n* = 637)	116 (29.7)	60 (24.4)	0.147
Stroke (*n* = 637)	113 (28.9)	60 (24.4)	0.213
Coronary artery disease (*n* = 303)	36 (20.6)	18 (14.1)	0.144
Arrhythmia (*n* = 292)	32 (19.5)	25 (19.5)	0.997
Valvular heart disease (*n* = 260)	16 (10.9)	9 (8.0)	0.429
Heart failure (*n* = 263)	14 (9.5)	10 (8.7)	0.831
Epilepsy (*n* = 233)	15 (11.6)	9 (8.7)	0.458
Migraine (*n* = 102)	17 (27.0)	14 (35.9)	0.342
**Cardiovascular risk factors:**	14 (9.5)	10 (8.7)	0.831
Hypertension (*n* = 421)	121 (50.2)	85 (47.2)	0.544
Hypercholesterolemia or use of statins (*n* = 395)	95 (43.0)	75 (43.1)	0.981
Diabetes mellitus (*n* = 417)	87 (36.7)	63 (35.0)	0.719
**Cardiovascular medication:**			
Antithrombotics (*n* = 939)	290 (48.7)	149 (43.4)	0.123
Other cardiovascular medication (*n* = 764)	253 (57.55)	178 (54.9)	0.480
**Call characteristics:**			
Median call duration in min:sec (IQR)	06:32 (04:43–08:54)	07:59 (05:54–10:50)	< 0.001
Median time for caller’s introduction in min:sec (IQR)	00:19 (00:12–00:27)	00:20 (00:13–00:29)	0.189
Initial call by someone else than the patient	621 (80.6)	342 (68.5)	< 0.001
Triage nurse consulted the general practitioner	449 (58.3)	305 (61.1)	0.319
**Main NTS complaint chosen by triage nurse**			
Neurological deficit	587 (76.2)	220 (44.1)	< 0.001
Dizziness	21 (2.7)	87 (17.4)	< 0.001
Headache	23 (3.0)	28 (5.6)	0.020
Odd behavior	21 (2.7)	18 (3.6)	0.375
Syncope	20 (2.6)	9 (1.8)	0.355
Vision problem	2 (0.3)	25 (5.0)	< 0.001^&^
Leg or arm problem	11 (1.4)	34 (6.8)	< 0.001
Other^^^	85 (11.0)	78 (15.6)	0.017
**Symptoms mentioned during the call**			
Decreased or loss of consciousness (*n* = 1103)	49 (7.4)	21 (4.8)	0.081
Face drooping (*n* = 713)	258 (54.3)	93 (39.1)	< 0.001
Arm weakness (*n* = 772)	254 (51.0)	77 (28.1)	< 0.001
Leg weakness (*n* = 653)	201 (49.0)	85 (35.0)	< 0.001
Sensory disturbances (*n* = 375)	192 (89.7)	150 (93.2)	0.243
Communication problem in general (*n* = 769)	413 (80.8)	187 (72.5)	0.008
Dysarthria (*n* = 416)	181 (65.1)	76 (55.1)	0.047
Dysphasia (*n* = 419)	163 (59.1)	72 (50.3)	0.089
Vision problem in general (*n* = 184)	68 (78.2)	82 (84.5)	0.266
Blurry vision (*n* = 74)	27 (77.1)	27 (69.2)	0.444
Diplopia (*n* = 74)	14 (63.6)	23 (44.2)	0.127
Reduced vision (*n* = 62)	15 (53.6)	22 (64.7)	0.374
Headache (*n* = 497)	147 (57.0)	140 (58.6)	0.718
Loss of balance/motor coordination (ataxia) (*n* = 236)	130 (86.1)	66 (77.6)	0.097
Dizziness (*n* = 312)	120 (82.2)	143 (86.1)	0.338
Seizure (*n* = 11)	4 (66.7)	3 (60.0)	0.819^&^
Short term memory loss (*n* = 68)	33 (76.7)	21 (84.0)	0.476
Shortness of breath (*n* = 403)	62 (24.4)	25 (16.8)	0.072
**Autonomic nervous system associated symptoms**			
Sweating (*n* = 208)	47 (36.7)	45 (56.3)	0.006
Nausea or vomiting (*n* = 311)	84 (61.8)	94 (53.7)	0.155
Pallor (*n* = 255)	54 (32.7)	27 (30.0)	0.655
Ashen skin (*n* = 198)	18 (14.1)	12 (17.1)	0.563
(Feeling of nearly) fainting (*n* = 1103)	57 (8.6)	41 (9.3)	0.680
**Course of symptoms**			
Onset of symptoms:			
Per acute (seconds) (*n* = 211)	52 (44.1)	56 (60.2)	0.020
Acute (minutes) (*n* = 211)	46 (39.0)	23 (24.7)	0.028
Gradually (hours) (*n* = 211)	20 (16.9)	14 (15.1)	0.710
Duration of symptoms ≤4.5 h (*n* = 986)	381 (61.4)	203 (55.6)	0.077
Symptoms still present at time of calling (*n* = 1254)	716 (93.4)	438 (89.9)	0.030
**Other characteristics**			
Caller expresses concern (*n* = 628)	334 (90.3)	248 (96.1)	0.006
Patient never experienced similar symptoms before (*n* = 368)	104 (49.8)	68 (42.8)	0.183
Recognition of symptoms:			
TIA (*n* = 368)	40 (19.1)	26 (16.4)	0.490
Stroke (*n* = 368)	25 (12.0)	16 (10.1)	0.566

*N* number (first column) stands for number of patients in which information on the variable of that row is known

*NTS* Netherlands Triage Standard, *IQR* interquartile range, *CVD* Cardiovascular disease, *TIA* Transient ischaemic attack

High NTS urgency: U1 and U2; Low NTS urgency: U3, U4 and U5

*Concerns all cardiovascular medication with the exception of antithrombotics; ^#^Pearson Chi Square Test for categorical variables and Mann-Whitney U Test for not normally distributed continuous variables; ^&^Fisher’s Exact Test for categorical variables; ^^^Amongst others: vomiting, dyspnea, neck symptoms, insult, disability problems (‘D from ABCD’)

The characteristics of patients with a known final diagnosis were comparable with those for whom the GP did not provide the final diagnosis (see Supplementary data Table S1).

Compared to the low NTS urgency group, patients in the high NTS urgency group were older (73.5 vs. 69.0 years, *p* < 0.001). Also, the call duration of patients in the high urgency group was shorter (06:32 min vs. 07:59 min, *p* < 0.001), and more often someone else called on behalf of the patient (80.6% vs. 68.5%, *p* < 0.001) in comparison to the low NTS urgency group. In nearly all calls concern about the symptoms was expressed (90.3% vs. 96.1%, *p* = 0.006), and in the vast majority, symptoms were still present at the time of calling (93.4% vs. 89.9%, *p* = 0.030). Patients classified as high urgent more often had face drooping (54.3% vs. 39.1%, *p* < 0.001), arm weakness (51.0% vs. 28.1%, *p* < 0.001), leg weakness (49.0% vs. 35%, *p* < 0.001), and communication problems in general (80.8% vs. 72.5%, *p* = 0.008), whereas patients classified as low urgent more often reported sweating (36.7% vs. 56.3%, *p* = 0.006).

### Final diagnoses

In 434 (34.2%) patients the final diagnosis was a TIA or minor stroke, and in 201 (15.8%) a major ischaemic or haemorrhagic stroke. Sixty-one (4.8%) patients had other LTEs, such as intracranial haemorrhage or meningitis. The remaining 573 patients (45.2%) were diagnosed with other neurological disorders (e.g. migraine, epilepsy) or other disorders (e.g. peripheral vestibular syndromes or psychogenic syndromes). See Table 3 for a complete overview of final diagnoses.

**Table 3:** Final diagnoses of 1269 patients who called the OHS-PC for symptoms suggestive of TIA/stroke

	High NTS urgency ***n*** = 770 (60.7%)	Low NTS urgency ***n*** = 499 (39.3%)	***P***-value
TIA/minor stroke	276 (35.8)	158 (31.7)	0.125
Major ischaemic or haemorrhagic stroke^a^	149 (19.4)	52 (10.4)	< 0.001
Other life threatening events (LTEs)^b^:	45 (5.8)	16 (3.2)	0.032
- Intracranial haemorrhage^c^	17 (37.8)	7 (43.8)	0.674
Migraine:	21 (2.7)	21 (4.2)	0.150
- With aura	9 (42.9)	7 (33.3)	0.525
Epilepsy	17 (2.2)	6 (1.2)	0.190
Syncope	18 (2.3)	12 (2.4)	0.939
Brain tumor	13 (1.7)	2 (0.4)	0.059^
Peripheral vestibular syndromes:	22 (2.9)	42 (8.4)	< 0.001
- Benign paroxysmal positional vertigo	10 (45.5)	11 (26.2)	0.119
- Meniere disease	1 (4.5)	1 (2.4)	0.999^
- Vestibular neuritis	0 (0.0)	5 (11.9)	0.155^
Peripheral nerve problem:	75 (9.7)	47 (9.4)	0.850
- Bell’s palsy	22 (29.3)	13 (27.7)	0.842
- Facial nerve palsy other than Bell’s palsy	53 (70.7)	34 (72.3)	0.842
Psychogenic syndromes	27 (3.5)	26 (5.2)	0.138
Other non-urgent diagnoses^d^	107 (13.9)	117 (23.4)	< 0.001

High NTS urgency: U1 and U2; Low NTS urgency: U3, U4 and U5. ^a^Including lacunar infarction and stroke not otherwise specified; ^b^Amongst others sepsis, acute coronary syndrome, meningitis, herpes encephalitis, coma, severe anemia due to gastrointestinal bleeding, hypoglycaemia, acute pulmonary embolism; ^c^Including subarachnoid haemorrhage; ^d^Amongst others guillain barre, multiple sclerosis, alcohol intoxication; ^Fisher’s Exact Test

### Final urgency allocation

**Fig. 2:**
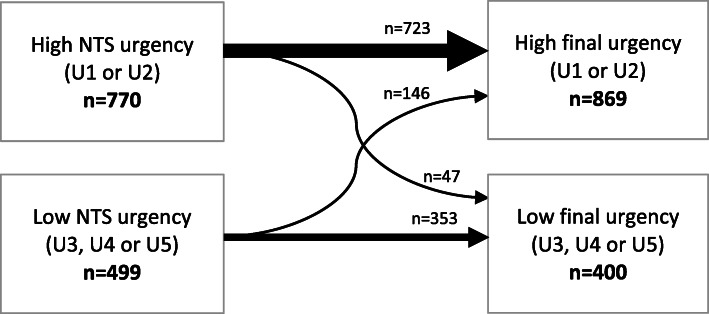
NTS urgency adjustments of 1269 patients with symptoms suggestive of TIA/minor stroke. This Figure does not show differences within the high and low urgency groups, for the differences within all urgency groups (U1-U5) see supplementary data Table S2

Of all 1269 patients, 770 (60.7%) received a high NTS urgency (U1 or U2) and 499 (39.3%) a low NTS urgency (U3, U4 or U5). In 728 (57.4%) patients the NTS urgency was equal to the final urgency. In the remaining 541 (42.6%) patients the NTS urgency was overruled, of which in 364 (67.3%) patients the NTS urgency was scaled up by the triage nurse, and in 177 (32.7%) patients it was scaled down (see Fig. 2 and supplementary data Table S2). Details on NTS urgency and final urgency specifically for patients with TIA/minor stroke only, for major stroke only, and for those with other LTEs only can be found in supplementary data Tables S3, S4 and S5.

### Accuracy of the NTS urgency and TIA/stroke, or TIA/stroke/other LTEs as the reference

The sensitivity of the NTS for allocating a high urgency to patients with TIA/stroke was 0.71 (95% CI 0.68–0.75), and for patients with TIA/stroke/other LTEs 0.72 (0.68–0.75). The specificity was 0.46 (0.42–0.50) and 0.48 (0.43–0.52), respectively. The positive and negative predictive values were 0.41 (0.38–0.43) and 0.75 (0.72–0.78) for TIA/stroke, and 0.62 (0.60–0.64) and 0.58 (0.54–0.62) for TIA/stroke/other LTEs, respectively.

### Accuracy of the final urgency (including overruling) and TIA/stroke, or TIA/stroke/other LTEs as the reference

The sensitivity of the final urgency allocation for allocating a high urgency to patients with TIA/stroke was 0.86 (0.84–0.89), and for TIA/stroke/other LTEs 0.86 (0.83–0.89). The specificity was 0.38 (0.34–0.42) and 0.40 (0.36–0.44), respectively. The positive and negative predictive values for TIA/stroke were 0.42 (0.40–0.44) and 0.84 (0.81–0.87), respectively, and for TIA/stroke/other LTEs 0.63 (0.62–0.65) and 0.70 (0.66–0.74), respectively. See also Table 4.

**Table 4:** Accuracy of adequate NTS urgency and final urgency allocation for detecting TIA/stroke/other LTEs

		Adequate NTS urgency allocation^**c**^ Value (95% CI)	Adequate final urgency allocation^**c**^ Value (95% CI)
**TIA/stroke**^**a**^	Sensitivity	0.71 (0.68–0.75)	0.86 (0.84–0.89)
	Specificity	0.46 (0.42–0.50)	0.38 (0.34–0.42)
	Positive predictive value	0.41 (0.38–0.43)	0.42 (0.40–0.44)
	Negative predictive value	0.75 (0.72–0.78)	0.84 (0.81–0.87)
**Other LTEs**^**b**^	Sensitivity	0.74 (0.61–0.84)	0.82 (0.70–0.91)
	Specificity	0.40 (0.37–0.43)	0.32 (0.30–0.35)
	Positive predictive value	0.06 (0.05–0.07)	0.06 (0.05–0.06)
	Negative predictive value	0.97 (0.95–0.98)	0.97 (0.95–0.98)
**TIA/stroke and other LTEs**	Sensitivity	0.72 (0.68–0.75)	0.86 (0.83–0.89)
	Specificity	0.48 (0.43–0.52)	0.40 (0.36–0.44)
	Positive predictive value	0.62 (0.60–0.64)	0.63 (0.62–0.65)
	Negative predictive value	0.58 (0.54–0.62)	0.70 (0.66–0.74)

^a^Prevalence TIA/minor stroke 34.2% and prevalence major stroke 15.8%; ^b^Prevalence other LTEs 4.8%; ^c^For TIA/minor stroke urgencies U1, U2 and U3 were all considered adequate, for major stroke and other LTEs urgencies U1 and U2 were considered adequate

## Discussion

### Summary

Of 1269 patients suspected of TIA/stroke, 635 (50.0%) showed to have a TIA or stroke; 434 (34.2%) had a TIA or minor stroke, 201 (15.8%) a major ischaemic or haemorrhagic stroke. In addition, 61 (4.8%) patients had other (neurologic) LTEs. The urgency allocation of the NTS tool was poor to moderate regarding sensitivity and specificity with TIA/stroke/other LTEs as the reference. In 42.6% the NTS urgency was overruled by the triage nurse. The final urgency allocation (including overruled NTS urgencies) showed modestly improved sensitivity (safety) whereas the specificity remained equally poor (efficiency). The positive predictive value did not change after overruling of the NTS, but the negative predictive value increased. This suggests that overruling by the triage nurses leads to safer telephone triage without compromising efficiency (i.e. overlapping confidence intervals of the NTS and final urgencies’ specificities).

### Strengths and limitations

This is the first study to report accuracy findings of the NTS tool for telephone triage at the OHS-PC with clinical outcomes as the reference. Because researchers were blinded to the final clinical outcome during data collection, the effect of hindsight bias was limited.

A limitation was missing data on the final clinical outcome (25% of all re-listened recordings). However, a detailed comparison in patient characteristics between those with a final outcome and those without showed that these groups were comparable (no indication of selection bias). Therefore, we believe our results are generalizable to similar OHS-PC settings.

### Comparison with existing literature

As described previously, many studies assessed the accuracy of other triage systems [22], and some of these also used clinical outcomes as the reference [24–31]. One study assessed the Manchester Triage System (MTS) in the domain of patients suspected for neurological disease seen at the ED. [32] The accuracy of a high urgency allocation was calculated with neurological disease (not otherwise specified) as the reference; a c-statistic of 0.73 was reported. High MTS urgency allocation was significantly associated with neurological disease (odds ratio 3.0, 95%CI 2.4–3.8, *p* < 0.001) [32]. Unfortunately, sensitivity or specificity was not calculated. Comparison to our study is also hampered, because in the primary care setting the prevalence of emergent cerebrovascular events is lower, and on average includes less severe cases. This may be reflected in less evident clinical presentations.

In addition to the studies on the accuracy evaluating all ‘main complaints’ of the triage systems, a few other studies described and evaluated diagnostic prediction models specifically for TIA and/or stroke in daytime general practice, namely: (i) the Dawson score, (ii) the modified Explicit Diagnostic Criteria for TIA (EDCT), and (iii) the TIA/stroke electronic decision support tool [39–41]. The Dawson score performed rather good for diagnosing TIA when validated in UK general practice, with a c-statistic of 0.70 (95% CI 0.66–0.75). However, sensitivity and specificity were not reported [39]. The modified EDCT criteria performed very good in Dutch daytime general practice with TIA/minor stroke as the reference, with a c-statistic of 0.86 (95% CI 0.80–0.92), a sensitivity of 0.98 (0.94–0.99) and a specificity of 0.74 (0.63–0.83) [40]. The accuracy of a TIA/ stroke electronic decision support tool in general practices in New Zealand was not reported, but the researchers reported that it did lead to improved triaging accuracy in the sense that it provided a widely applicable and cost-effective way of improving care and outcomes for patients with TIA/stroke [41]. Importantly, however, comparison of our results to the previous studies on diagnostic prediction models for TIA in daytime general practice is limited, because these studies included only patients with resolved symptoms, which is in contrast to our primary care population calling the OHS-PC; 90.9% of all patients had symptoms when calling.

In our study, we considered different urgency levels as adequate; for TIA/minor stroke U1-U3, and for major stroke/other LTEs U1-U2. The rationale for high urgency allocations in suspected stroke patients is mainly because of available treatment options, and not because TIA/stroke may result in ABCD instability (i.e. airway, breathing, circulation, disability). Assigning high urgency levels to patients with acute stroke enables early initiation of (invasive) prognostically beneficial treatment [42–44]. In patients with TIA/minor stroke early initiation of antiplatelets for secondary stroke prevention is key, given the substantial risk of major stroke in the first hours to days after a TIA [5, 6, 45]. Current treatment guidelines on TIA/stroke recommend that patients suspected of TIA should be seen within 24 h after symptom onset at a TIA outpatient clinic for a neurological assessment, while secondary stroke prevention should be started as soon as possible after a confirmed diagnosis of TIA/minor stroke [46, 47] or directly if the patient cannot be assessed by a neurologist the same day [48]. Therefore, we considered U3 (patient seen within 3 h) as sufficient in patients who finally showed to have had a TIA/minor stroke.

### Implications for research and/or practice

Our study indicated that the accuracy of the NTS was poor to moderate, yet safety improved after overruling by the triage nurse. Apparently, triage nurses and/or their GP supervisors capture some vital patient information that is not yet incorporated in the NTS. Further improvement of safety, as well as improving efficiency of telephone triage in the domain of patients calling with neurological symptoms is necessary. Improving the accuracy of already existing triage systems such as the NTS should be the first step. In order to do so, prediction models are needed based on multivariable analyses to provide an evidence-based basis for which triage questions are helpful, and which are not.

## Conclusions

The NTS decision support tool used in Dutch OHS-PC performed poor to moderately regarding safety (sensitivity) and efficiency (specificity) in allocating adequate urgencies to patients with and without TIA/stroke/other LTEs. There are indications that overruling the NTS by triage nurses improves safety, without compromising efficiency.

## Supplementary Information

**Additional file 1: Table S1.** Baseline characteristics of 1,700 patients with symptoms suggestive of TIA/stroke, classified into patients of whom follow-up information about the final diagnosis could and could not be retrieved. **Table S2.** NTS urgency and final urgency allocation of 1,269 patients with symptoms suggestive of TIA/minor stroke. **Table S3.** NTS urgency and final urgency allocation of 434 patients with TIA/minor stroke. **Table S4.** NTS urgency and final urgency allocation of 201 patients with major stroke. **Table S5.** NTS urgency and final urgency allocation of 61 patients with other LTEs.

## Data Availability

The datasets generated during and/or analysed during the current study are available from the corresponding author on reasonable request.

## Abbreviations

GP: General Practitioner
LTE: Life-Threatening Events
NTS: Netherlands Triage Standard
OHS-PC: Out-Of-Hours Services in Primary Care
TIA: Transient Ischaemic Attack
