# Supplementary material for: Accuracy of telephone triage in patients suspected of transient ischaemic attack or stroke: a cross-sectional study
Source: BMC Fam Pract. 2020 Dec 5;21:256. doi: 10.1186/s12875-020-01334-3 (PMC7719259; doi:10.1186/s12875-020-01334-3)
Supplement: Supplementary file 1 — Additional file 1: Table S1. Baseline characteristics of 1,700 patients with symptoms suggestive of TIA/stroke, classified into patients of whom follow-up information about the final diagnosis could and could not be retrieved. Table S2. NTS urgency and final urgency allocation of 1,269 patients with symptoms suggestive of TIA/minor stroke. Table S3. NTS urgency and final urgency allocation of 434 patients with TIA/minor stroke. Table S4. NTS urgency and final urgency allocation of 201 patients with major stroke. Table S5. NTS urgency and final urgency allocation of 61 patients with other LTEs. [file 12875_2020_1334_MOESM1_ESM.pdf]

Supplementary data

Table S1. Baseline characteristics of 1,700 patients with symptoms suggestive of TIA/stroke, classified into patients of whom follow-up information about the final diagnosis could and could not be retrieved.

	**Final diagnosis available n=1269 (74.6%)**	**Final diagnosis not available n=431 (25.4%)**	**P-value[#]**
**Patient characteristics**			
Median age in years (IQR)	72.0 (57.0-83.0)	73.0 (58.0-84.0)	0.495
Female sex	722 (56.9)	239 (55.5)	0.602
Family history of CVD (n=41)	27 (75.0)	3 (60.0)	0.478[&]
**Medical history:**			
Cardiovascular disease (n=1181)	680 (77.1)	253 (84.6)	0.006
TIA (n=853)	176 (27.6)	65 (30.1)	0.483
Stroke (n=853)	173 (27.2)	73 (33.8)	0.063
Coronary artery disease (n=394)	54 (17.8)	28 (30.8)	0.008
Arrhythmia (n=375)	57 (19.5)	21 (25.3)	0.252
Valvular heart disease (n=331)	25 (9.6)	9 (12.7)	0.452
Heart failure (n=336)	24 (9.1)	10 (13.7)	0.252
Epilepsy (n=294)	24 (10.3)	8 (13.1)	0.530
Migraine (n=134)	31 (30.4)	10 (31.3)	0.927
**Cardiovascular risk factors:**			
Hypertension (n=559)	206 (48.9)	79 (57.2)	0.090
Hypercholesterolemia or use of statins (n=497)	170 (43.0)	46 (45.1)	0.708
Diabetes mellitus (n=538)	150 (36.0)	58 (47.9)	0.017
**Cardiovascular medication:**			
Antithrombotics use (n=1259)	439 (46.8)	165 (51.6)	0.137
Other cardiovascular medication (n=1019)	431 (56.4)	161 (63.1)	0.060
**Call characteristics**			
Median call duration in min:sec (IQR)	07:08 (05:07-09:37)	07:26 (05:10-09:42)	0.350
Median time for caller's introduction in min:sec (IQR)	00:20 (00:13-00:28)	00:19 (00:12-00:27)	0.744
Initial call by someone else than the patient	963 (75.9)	324 (75.2)	0.766
Triage nurse consulted the general practitioner	754 (59.4)	259 (60.1)	0.805
**Main NTS complaint chosen by triage nurse**			
Neurological deficit	807 (63.6)	292 (67.7)	0.119
Dizziness	108 (8.5)	23 (5.3)	0.033
Headache	51 (4.0)	13 (3.0)	0.345
Odd behavior	39 (3.1)	14 (3.2)	0.857
Syncope	29 (2.3)	6 (1.4)	0.259
Vision problem	27 (2.1)	13 (3.0)	0.293
Leg or arm problem	45 (3.5)	18 (4.2)	0.550
Other[^]	163 (12.8)	52 (12.1)	0.674
**Symptoms mentioned during the call**			
Decreased or loss of consciousness (n=1467)	70 (6.3)	19 (5.2)	0.435
Face drooping (n=959)	351 (49.2)	112 (45.5)	0.317
Arm weakness (n=1060)	331 (42.9)	123 (42.7)	0.961
Leg weakness (n=890)	286 (43.8)	105 (44.3)	0.893
Sensory disturbances (n=496)	342 (91.2)	109 (90.1)	0.710
Communication problem in general (n=1033)	600 (78.0)	211 (79.9)	0.517
Dysarthria (n=554)	257 (61.8)	78 (56.5)	0.274
Dysphasia (n=559)	235 (56.1)	74 (52.9)	0.506

Vision problem in general (n=250)	150 (81.5)	50 (75.8)	0.315
Blurry vision (n=96)	54 (73.0)	11 (50.0)	0.043
Diplopia (n=93)	37 (50.0)	9 (47.4)	0.838
Vision loss (n=82)	37 (59.7)	14 (70.0)	0.408
Headache (n=644)	287 (57.7)	93 (63.3)	0.232
Loss of balance/motor coordination (ataxia) (n=298)	196 (83.1)	55 (88.7)	0.277
Dizziness (n=396)	263 (84.3)	64 (76.2)	0.082
Seizure (n=15)	7 (63.6)	2 (50.0)	0.999[&]
Short term memory loss (n=90)	54 (79.4)	14 (63.6)	0.135
Shortness of breath (n=535)	87 (21.6)	25 (18.9)	0.516
**Autonomic nervous system associated symptoms**			
Sweating (n=278)	92 (44.2)	29 (41.4)	0.683
Nausea or vomiting (n=399)	178 (57.2)	40 (45.5)	0.050
Pallor (n=343)	81 (31.8)	26 (29.5)	0.698
Ashen skin (n=271)	30 (15.2)	11 (15.1)	0.986
(Feeling of nearly) fainting (n=1467)	98 (8.9)	31 (8.5)	0.830
**Course of symptoms**			
Onset of symptoms:			
Per acute (seconds) (n=272)	108 (51.2)	31 (50.8)	0.960
Acute (minutes) (n=272)	69 (32.7)	23 (37.7)	0.467
Gradually (hours) (n=272)	34 (16.1)	7 (11.5)	0.373
Duration of symptoms ≤4.5 hours (n=1317)	584 (59.2)	215 (65.0)	0.065
Symptoms still present at time of calling (n=1676)	962 (93.5)	585 (90.4)	0.022
**Other characteristics**			
Caller expresses concern (n=818)	582 (92.7)	179 (94.2)	0.466
Patient never experienced similar symptoms before (n=501)	172 (46.7)	59 (44.4)	0.637
Recognition of symptoms:			
TIA (n=501)	66 (17.9)	24 (18.0)	0.977
Stroke (n=501)	41 (11.1)	22 (16.5)	0.107

NTS: Netherlands Triage Standard; IQR: interquartile range; CVD: cardiovascular disease; TIA: transient ischaemic attack. *Concerns all cardiovascular medication with the exception of antithrombotics; [#]Pearson Chi Square Test for categorical variables and Mann-Whitney U Test for not normally distributed continuous variables; [&]Fisher's Exact Test for categorical variables; [^]Amongst others: vomiting, dyspnea, neck symptoms, insult, disability problems ('D from ABCD').

Table S2. NTS urgency and final urgency allocation of 1,269 patients with symptoms suggestive of TIA/minor stroke

	Final U1	Final U2	Final U3	Final U4	Final U5	Total
**NTS U1**	229	115	8	1	7	360
**NTS U2**	27	352	11	10	10	410
**NTS U3**	1	14	92	7	7	121
**NTS U4**	0	7	9	15	1	32
**NTS U5**	41	83	123	59	40	346
**Total**	298	571	243	92	65	1269

Table S3. NTS urgency and final urgency allocation of 434 patients with TIA/minor stroke

	Final U1	Final U2	Final U3	Final U4	Final U5	Total
**NTS U1**	71	45	3	1	3	123
**NTS U2**	10	131	3	3	6	153
**NTS U3**	1	1	24	2	0	28
**NTS U4**	0	1	3	0	0	4
**NTS U5**	18	35	41	20	12	126
**Total**	100	213	74	26	21	434

Table S4. NTS urgency and final urgency allocation of 201 patients with major stroke

	Final U1	Final U2	Final U3	Final U4	Final U5	Total
**NTS U1**	58	25	2	0	1	86
**NTS U2**	8	49	3	1	2	63
**NTS U3**	0	1	11	0	0	12
**NTS U4**	0	0	0	2	0	2
**NTS U5**	6	15	11	5	1	38
**Total**	72	90	27	8	4	201

Table S5. NTS urgency and final urgency allocation of 61 patients with other LTEs

	Final U1	Final U2	Final U3	Final U4	Final U5	Total
**NTS U1**	23	7	0	0	0	30
**NTS U2**	2	13	0	0	0	15
**NTS U3**	0	0	2	0	0	2
**NTS U4**	0	0	1	2	0	3
**NTS U5**	3	2	5	1	0	11
**Total**	28	22	8	3	0	61
